# Appropriate approach to technology in animal hematology

**DOI:** 10.1016/j.toxrep.2018.12.008

**Published:** 2018-12-31

**Authors:** Javad Beheshtipour

**Affiliations:** Young Researchers and Elite Club, Sanandaj Branch, Islamic Azad University, Sanandaj, Iran

**Keywords:** Animal, Hematology, Human

Dear Editor,

I studied Jacob et al. article carefully, entitled “Oral toxicity study of sports nutritional powder in Wistar rats: A 90 day repeated dose study”, published in this journal (Vol, 5, pp. 497–503) [1]. It is interesting to note that this scientific subject has been dealt with, but one point in the article is the focus of my attention.

The foundation of the Sysmex KX-21 hematology analyzer is based on the three axes (Fig. 1): First, creating the proper concentration of human red blood cells (RBCs) in order to prevent piles up blood cells. Second, the presence of an aperture based on the diameter of human RBCs to pass one by one, of the cells, and thirdly, the generated electrical resistance by embedding electrodes in the aperture when passing RBCs [2].

**Fig. 1:**
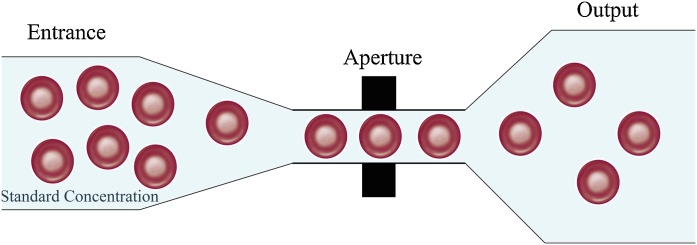
Schematic representation of the performance of Sysmex KX-21 hematology analyzer.

Human RBCs are approximately 7.5 to 8.7 μm in diameter [3], whereas rat RBCs are 6 to 7 μm [4]. Hence, device capabilities to be challenged in confronting with rat RBCs in all axes. This means that the created concentration by the device (standardized for human RBCs) changes the distance between the rat RBCs. In the following, the RBCs are transmitted at irregular intervals within aperture over a specified period (standardized for human) simultaneously with the produced electrical resistance by the passage of the erythrocytes (the distance between the electrodes is founded on the human RBC diameter). Finally, these events lead to mistakes in accurately counting of rat RBCs in the Sysmex KX-21 hematology analyzer. The count methods of the platelets (PLT) and white blood cells (WBCs) are similar to the RBCs. Thus, PLT and WBCs counting are also impaired. It should be noted that for WBCs counting, the red cells are lysed by the stromatolyser solution of the device [5,6].

Hematocrit (HCT) is obtained using impedance technology whereby the passage of each individual cell through the aperture generates an electrical pulse that is assumed to be proportional to the volume of the cell [7,8]. The HCT on a Sysmex KX-21 hematology analyzer is acquired using the cumulative pulse. Therefore, according to the principles of machine function, HCT is affected too.

Mean corpuscular volume (MCV), mean cell hemoglobin (MCH), and mean cell haemoglobin concentration (MCHC) are calculated by a hematology analyzer via the following formulas [9]:

MCV (fl) = HCT/RBC

MCH (pg) = hemoglobin/RBC

MCHC (g/dl) = hemoglobin/HCT

Due to the calculated values of HCT and RBC by the device, the above parameters are impacted.

In Jacob et al. paper, hematological parameters in rats were measured by human's automated hematology analyzer (Sysmex, KX-21) which is false due to the different sizes of human RBCs, WBCs, and PLT with rodents [3,4].

## Conflict of interest

None.
